# The EuroBioBank Network: 10 years of hands-on experience of collaborative, transnational biobanking for rare diseases

**DOI:** 10.1038/ejhg.2014.272

**Published:** 2014-12-24

**Authors:** Marina Mora, Corrado Angelini, Fabrizia Bignami, Anne-Mary Bodin, Marco Crimi, Jeanne- Hélène Di Donato, Alex Felice, Cécile Jaeger, Veronika Karcagi, Yann LeCam, Stephen Lynn, Marija Meznaric, Maurizio Moggio, Lucia Monaco, Luisa Politano, Manuel Posada de la Paz, Safaa Saker, Peter Schneiderat, Monica Ensini, Barbara Garavaglia, David Gurwitz, Diana Johnson, Francesco Muntoni, Jack Puymirat, Mojgan Reza, Thomas Voit, Chiara Baldo, Franca Dagna Bricarelli, Stefano Goldwurm, Giuseppe Merla, Elena Pegoraro, Alessandra Renieri, Kurt Zatloukal, Mirella Filocamo, Hanns Lochmüller

**Affiliations:** 1 Muscle Cell Biology Lab, Neuromuscular Diseases and Neuroimmunolgy Unit, Fondazione Istituto Neurologico C. Besta, Milano, Italy; 2 IRCCS Fondazione San Camillo Hospital, Lido Venice, Italy; 3 Department of Neurosciences, NPSRR University of Padova, Padova, Italy; 4 GlaxoSmithKline, London, UK; 5 EURORDIS, European Organisation for Rare Diseases, Paris, France; 6 Fondazione Telethon, Milan, Italy; 7 3 C-R, Castelginest, France; 8 Laboratory of Molecular Genetics and Malta BioBank, University of Malta, and Thalassaemia Clinic, Mater Dei Hospital, Msida, Malta; 9 Hôpital Foch, Suresnes, France; 10 Department of Molecular Genetics and Diagnostics, National Institute of Environmental Health, Budapest, Hungary; 11 MRC Centre for Neuromuscular Diseases, Institute of Genetic Medicine, Newcastle University, International Centre for Life, Newcastle upon Tyne, UK; 12 Faculty of Medicine, University of Ljubljana, Ljubljana, Slovenia; 13 Neuromuscular Unit, IRCCS Foundation Ca' Granda Ospedale Maggiore Policlinico, Dino Ferrari Centre, University of Milan, Milan, Italy; 14 Division of Cardiomyology and Medical Genetics, Department of Experimental Medicine, Second University of Naples, Naples, Italy; 15 Manuel Posada de la Paz, Institute of Rare Diseases Research, IIER, ISCIII and Spain RDR & CIBERER, Madrid, Spain; 16 GENETHON, DNA and Cell Bank, Evry, France; 17 Muscle Tissue Culture Collection, Friedrich-Baur-Institute, Neurological Department, Ludwig-Maximilians-University of Munich, Munich, Germany; 18 Molecular Neurogenetics Unit, Fondazione Istituto Neurologico C. Besta, Milano, Italy; 19 Department of Human Molecular Genetics and Biochemistry, Sackler Faculty of Medicine, Tel-Aviv University, Tel-Aviv, Israel; 20 Dubowitz Neuromuscular Centre, MRC Neuromuscular Centre at UCL Institute of Child Health, London, UK; 21 Department of Human Genetics, Centre Hospitalier Universitaire de Quebec, Quebec City, Quebec, Canada; 22 Inserm U974—Institute of Myology, University Pierre and Marie Curie Paris 6, Paris, France; 23 Laboratorio di Genetica Umana, E.O. Ospedali Galliera, Genova, Italy; 24 Dipartimento Ligure di Genetica, E.O. Ospedali Galliera, Genova, Italy; 25 Parkinson Institute, Istituti Clinici di Perfezionamento, Milano, Italy; 26 Medical Genetics Unit, IRCCS Casa Sollievo della Sofferenza Hospital, San Giovanni Rotondo, Italy; 27 Division of Medical Genetics, University of Siena, Azienda Ospedaliera Universitaria Senese, Siena, Italy; 28 Institute of Pathology, Medical University of Graz, Graz, Austria; 29 Centro di Diagnostica Genetica e Biochimica delle Malattie Metaboliche, Istituto G. Gaslini, Genova, Italy

## Abstract

The EuroBioBank (EBB) network (www.eurobiobank.org) is the first operating network of biobanks in Europe to provide human DNA, cell and tissue samples as a service to the scientific community conducting research on rare diseases (RDs). The EBB was established in 2001 to facilitate access to RD biospecimens and associated data; it obtained funding from the European Commission in 2002 (5th framework programme) and started operation in 2003. The set-up phase, during the EC funding period 2003–2006, established the basis for running the network; the following consolidation phase has seen the growth of the network through the joining of new partners, better network cohesion, improved coordination of activities, and the development of a quality-control system. During this phase the network participated in the EC-funded TREAT-NMD programme and was involved in planning of the European Biobanking and Biomolecular Resources Research Infrastructure. Recently, EBB became a partner of RD-Connect, an FP7 EU programme aimed at linking RD biobanks, registries, and bioinformatics data. Within RD-Connect, EBB contributes expertise, promotes high professional standards, and best practices in RD biobanking, is implementing integration with RD patient registries and ‘omics' data, thus challenging the fragmentation of international cooperation on the field.

## Introduction

The Organisation for Economic Co-Operation and Development (OECD) defines a biobank as ‘A collection of biological material and the associated data and information stored in an organised system, for a population or a large subset of a population'.^1^ The collection of biological material and data for research and diagnosis has a long history in educational and medical institutions. In the past, biorepositories tended to be inconspicuous—the responsibility of individual research groups or institutions, and biospecimens, were rarely shared with other laboratories. With recent technological advances, biorepositories are being opened up for new uses (when permitted by national regulations), and new biorepositories are being established as part of funded, but time-limited, research projects, whereas information technology now enables the systematic linkage and tracking of samples and data, and has provided tools for access and analysis across vast sample sets and data sets.

In the field of rare diseases (RDs) the number of available biospecimens is, in general, very limited. As a direct consequence of disease rarity, clinical trials are difficult to perform and so a limited number of treatments have been developed, whereas disease prognosis and natural history are poorly known, and patients with RDs do not receive the care and medical attention available to people with common diseases. Sharing material and data on RDs is essential for identifying disease-causing genes, studying pathological mechanisms, and developing treatments.

In order to improve the accessibility of biospecimens and associated data on RDs, the EuroBioBank (EBB) network, involving 16 partners from eight European countries (Belgium, France, Germany, Hungary, Italy, Malta, Slovenia, and Spain), was established in 2001. The EBB obtained funding from the European Commission in 2002 (5th framework programme; EuroBioBank project QLRI-CT-2002-02769) and started work in 2003.

This report describes the development of the EBB network over the past decade, its achievements, and the major challenges it has already faced and expects to face in the future.

## Set-up phase

The EBB network (www.eurobiobank.org) was the first operating network of biobanks in Europe to provide human DNA, cell, and tissue samples as a service to the scientific community conducting research on RDs. The idea of a network was first promoted by two patient organisations: the Association Française contre les Myopathies (AFM) and the European Organisation for RDs (EURORDIS). These organisations took cognisance of various circumstances and events, which made it evident that a supranational biobank network was necessary, these included the following: letters from families and patients with RDs offering blood or other biological material to further RD research; scientific publications noting that difficulties in obtaining biological material from ‘informative' RD families were holding back research; the need to avoid wasting or loosing samples particularly when a research project wound down; the expansion of genomic research that raised hopes of earlier and more accurate diagnoses as well as more effective treatments; the creation of two biobanks—Généthon and Myobank-AFM (formerly Banque de Tissus pour la Recherche); and the establishment of French and European biobank networks for common diseases.

A meeting to set up the EBB was held in Paris in 2001 with 16 founding partners (Table 1). At this time (1998–2002) the European 5th Framework Programme for Research and Development entitled ‘Quality of Life and Management of Living Resources' was soliciting applications for funding. A proposal was submitted under action line 14.1 ‘Support for Research Infrastructures' and was accepted (Proposal No. QLRI-CT-2002-02769). A total of 1.22 M € was made available to the nascent EBB network by the European Commission for 36 months, starting 1 January 2003. The project period was subsequently extended for a further 3 months (to 31 March 2006). EURORDIS, a patient-driven European organisation for RDs, administered the EBB network from then to 2011.

The aims of the EBB network were to identify and locate repositories of biological material (DNA, tissues, and cell cultures) pertaining to RDs, to harmonise and disseminate quality banking practices, to distribute quality material and associated data to scientific users, and to disseminate biobank-pertinent knowledge and know-how to the scientific community through specialised training courses, conferences, articles, and a website.

Expected achievements included reorganization of existing bio-collections; improvement of medical and scientific collaboration in the field of RDs; and encouragement of research with concomitant development of new diagnostic tools and therapies for RDs.

The project was organized into work packages (Table 2). The scientific coordinator was Dr Cécile Jaeger of AFM, and the administrative coordinator was Dr Fabrizia Bignami of EURORDIS. Dr Jaeger retired from the project in 2005 and Dr Hanns Lochmüller (then of the University of Munich, Germany; now at the Newcastle University, UK) took over as scientific coordinator until 2012 (with annual re-elections from 2005 to 2011), when he resigned and Dr Marina Mora of the Besta Neurological Institute, Milan, was elected by the EBB general assembly.

## Consolidation phase and TREAT-NMD

**Figure 1:**
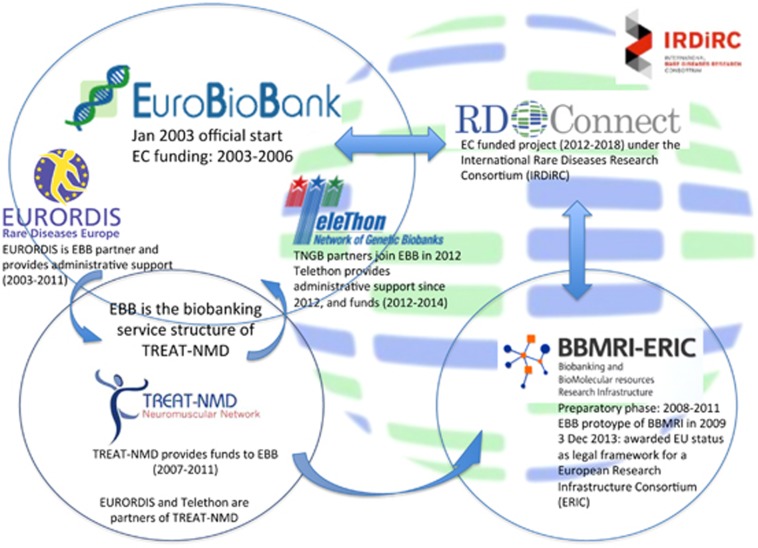
Scheme illustrating interactions of EBB with European organisations and networks. Coordination of RD-Connect is lead by Hanns Lochmüller, who has also been the coordinator of TREAT-NMD; coordinator of the Telethon Network of Genetic Biobanks is Mirella Filocamo; coordinator of BBMRI during the preparatory phase was Kurt Zatloukal, now BBMRI-ERIC has a general director which is Jan-Eric Litton; EURORDIS is coordinated by a Board of Directors composed of elected RD patient organization representatives from countries around Europe (www.eurordis.org); IRDiRC is governed by an executive committee, three scientific committees and a number of working groups (www.irdirc.org).

When the EC-funded period expired (31 March 2006), EBB was kept going with funds contributed by each partner for 1 year in order to maintain the catalogue functional, meet annually, and seek further funding. In 2007, EBB became part of the TREAT-NMD project in order to carry out biobanking for translational research in neuromuscular disorders (Figure 1). TREAT-NMD is a network of excellence funded by the EC (framework programme 6, 2007–2011) to provide an infrastructure (including biobanking) promoting the transition of promising new treatments for patients with neuromuscular diseases from preclinical development to clinical practice, and to establish best-practice care for patients with these diseases. This network, after EC funding expired, has developed from its European roots to become a global organization, the TREAT-NMD Alliance, bringing together leading specialists, patient groups, and industry representatives to ensure readiness for the clinical trials and therapies for the future, while promoting best practices today.

In 2010, TREAT-NMD held a public consultation inviting all stakeholders to provide feedback on the impact of the TREAT-NMD activities and to provide guidance on the future priorities. The activity of the TREAT-NMD Network that received the highest recommendation—with over 90% indicating this as a top priority for the Network, was that of facilitating international collaborations to share data, experience and develop harmonised tools and protocols, and this included biobanking activities (http://www.treat-nmd.eu/downloads/file/consultation/TREAT-NMD_Consultation_Document_Sept2010.pdf).

From 2012, the Fondazione Telethon—partner of TREAT-NMD—took on the responsibility for EBB as a 3-year commitment within the newly established TREAT-NMD Alliance. Fondazione Telethon was already supporting genetic biobanks in Italy since 1993. In 2008, Telethon had unified all its biobanks, creating the Telethon Network of Genetic Biobanks (TNGB) biobanknetwork.telethon.it, the first of this kind in Italy, with a virtual catalogue of biospecimens and associated data that presently lists more than 750 rare defects.^2^ In 2012, the natural progression has been to join the two Networks, EBB and TNGB. Of note, all neuromuscular biobanks of TNGB were already EBB members. A total of 10 additional partners have joined the EBB network since 2007 making to date a total of 21 biobanks plus 4 non-biobank members (Table 1). Of note, biobanks from three additional countries (UK, Canada, and Israel) have been accepted.

From 2007, on the network only pays for joint services such as the website, the catalogue updating and annual meetings; while all the EBB partners had to cover their own costs regarding the operation of the biobanks either with institutional funding or with specific grants (such as Telethon grants supporting TNGB).

## BBMRI-ERIC

As part of TREAT-NMD, EBB collaborated with the European Biobanking and Biomolecular Resources Research Infrastructure (implemented under the European Research Infrastructure Consortium; BBMRI-ERIC), in the planning of which it was represented by EURORDIS and by individual EBB biobanks that were full or associated partners in the BBMRI preparatory phase. EBB's participation in BBMRI was somewhat uncertain at the beginning as the latter had no specific strategy for RDs and RD biobanking (BBMRI originally came from big population biobanking). However, BBMRI was open to the argument by the EBB coordinators, that biobanks with less than 100 000 samples should be considered and might be extremely useful for research, particularly in RD. EBB eventually became part of the BBMRI Prototype in August 2009, and BBMRI and BBMRI-ERIC partner thanks to the FP7-funded project RD-Connect. BBMRI-ERIC is an infrastructure with sustainable funding from European member states covering a wide area of biobanks including bioresources for all diseases, irrespective of whether they are considered common or rare, as well as population-based cohort studies.^3, 4, 5^ However, it was acknowledged that RD research presents specific opportunities and challenges that require specific procedures and distinguish RD biobanking from other forms of biobanking.^6, 7^ For this reason BBMRI-ERIC is considering the establishment of a common service for RD to specifically address issues related to RD biobanks.

## Governance

The EuroBioBank Network Charter has been the constituting instrument of EBB from January 2006. It sets out the principles agreed upon by the partners of the network. In particular, the Charter recalls the ethical guidelines endorsed by the EBB Network, defines the organisation and governance of the EBB, establishes the benefits and duties attached to partnership, as well as the conditions of access to and withdrawal of partnership.

The EBB General Assembly is the decision-making and arbitration body of the Network that takes decisions on: (i) strategic orientations of EBB; (ii) establishment of an annual work plan and setting up of working groups; (iii) modifications and amendments to the EBB organisation and the Network Charter and appointment of the scientific coordinator; (iv) the EBB budget, management (partnership fees, investments, and so on) in collaboration with the finance manager responsible for the EBB account; (v) terms of use of the EBB name and logo; (vi) inclusion of new partners and exclusion of a partner; and (vii) approval of each single sample request from private for-profit organizations.

### Membership

The entry of new, European and non-European biobanks into the EBB network is encouraged. To this end, an evaluation procedure and specific assessment criteria have been established. Such criteria ensure adherence to minimum entry conditions that include the following: presence of collections of RD biological samples and their availability to the scientific community, a quality-control (QC) system in place for the management of the biobank, with standard operating procedures (SOPs) regulating sample and data acquisition, and sample processing, storage, and distribution. The candidate biobanks should also adhere to Ethical, Legal and Social Implication (ELSI) principles and comply with the recommandations issued by the Oviedo Convention and the OECD Task Force on Biological Resource Centers,^1, 8^ and with the national and European laws and regulations.

## Current access to samples

Biobanks and biomaterial collections across the world can join EBB. The member biobank maintains the legal custodianship of samples, whereas the EBB acts as a clearing house or ‘virtual' biobank with its online catalogue and search engine for locating samples. Researchers from anywhere in the world who locate a sample of interest through the catalogue can directly contact the biobank holding the sample. Sample distribution is governed by the conditions set out in the EBB charter and standardised material transfer agreements (MTAs).

## Achievements and recognition

In addition to complying with workpackage tasks (Table 2), the EEB has prepared 29 SOPs pertaining to sample and data acquisition, sample processing and storage, and sample distribution; has published (2005) a book on ethical and legal issues concerning international biobanking;^9^ has been awarded the Newropeans Grand Prix 2004 for Research and Technology; has been cited as a ‘European model of coordination and of integration of Biological Resource Centres for the optimisation and improvement of the use of human biomaterial at European level', in 2006, by the Institute for Prospective Technological Studies/European Science and Technology Observatory network.

An immediate outcome of the effort to establish the EBB network was the improved functioning of each EBB biobank member. Each biobank was forced to better organize the data to be included into the catalogue and to harmonize SOPs. This was facilitated by personnel training provided by the EBB organization, and by exchange of solutions within the network, including sharing rules for sample distribution and network acknowledgement in publications making use of EBB-supplied samples. Being part of the network, with specific agreed rights and duties under the network's governance model, also contributed to improvement for the individual biobanks, for example, systematically recording biobanking activities, adhering to SOPs, and complying with ELSI principles.

### The catalogue

A great deal of work was devoted to drawing the EBB catalogue, in particular to define: catalogue content, vocabulary lists used for the minimum data set (normalized terms), how to update the catalogue, and how to search in the catalogue. The partners agreed on the following minimum data set: (i) type of sample, (ii) classification of the disease based on ICD-10 identifier and name, MIM number and name, (iii) number of families, (iv) number of patients, (v) anatomic origin, and (vi) biobank contact. As 2011 the catalogue was further implemented by the addition of an optional field for the ORPHA code, specially developed by Orphanet (www.orpha.net), as commonly used coding systems, like ICD or SNOMED CT codes, do not cover most of the RD names.

**Figure 2:**
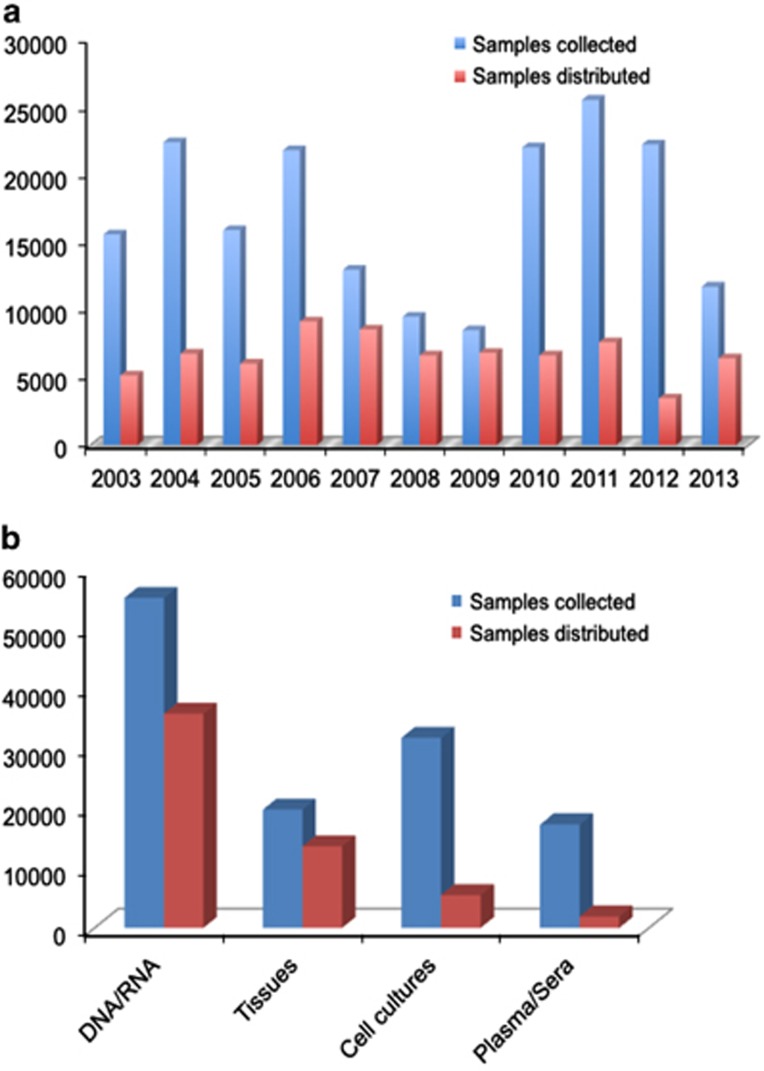
Histograms showing (**a**) number of samples collected and distributed by the EBB network during period 2003–2013; and (**b**) type of biomaterials collected and distributed in total.

The web-based EBB catalogue (http://www.eurobiobank.org/en/services/CatalogueHome.html) makes it possible to search for biological samples by type of biological material and disease. Of more than 500 000 samples stored in the EBB biobanks, 130 000 were available on the catalogue at the end of 2013; 188 400 new samples were collected from 2003 to 2013 and 73 400 samples were distributed over the same period. On average, 18800 samples (5700 neuromuscular disease samples, NMD) are collected and 7000 samples distributed (3000 NMD samples) each year (Figure 2).

### Research publications

**Figure 3:**
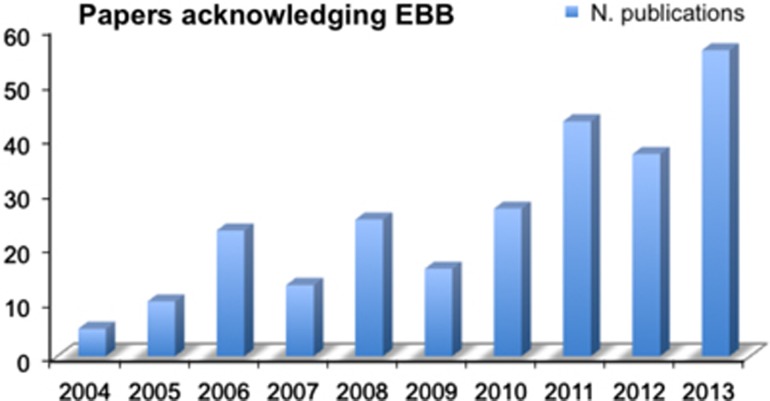
Histogram showing numbers of papers acknowledging EBB during the period 2004–2013.

Up to December 2013, 255 original articles of research specifying the use of EBB biospecimens had been published (Figure 3).

DNA samples were used mainly for diagnosis, for molecular-genetic studies to identify new disease-related genes, for studying human historical migrations, and to characterize epigenetic factors affecting disease phenotype.^10, 11, 12, 13, 14, 15, 16, 17, 18, 19, 20, 21, 22^

Studies on EBB cell samples have been concerned with issues such as elucidation of biological pathways involved in diseases, *in vitro* characterization of muscle–immune interactions, molecular analysis of DNA methylation, chromatin structure, and epigenetic factors affecting disease expression. Cells were used in gene transfection and gene-silencing experiments, in cell stimulation with growth factors and cytokines, to investigate exon skipping with antisense oligonucleotide treatment, to develop *in vitro* models for drug screening, to study mechanisms of muscular dystrophies with assays such as membrane fusion, cell migration, immunochemistry, cell surface protein clustering, and viral-mediated protein expression.^23, 24, 25, 26, 27, 28, 29, 30, 31, 32, 33, 34^

Tissue samples were used to search for disease biomarkers, to define histopathological features of diseases, to verify protein expression and enzyme activity, and to obtain RNA for authentication of variants affecting RNA splicing.^35, 36, 37, 38, 39, 40, 41, 42^ Serum and plasma samples were used in biomarker discovery and validation, in particular by the EC-funded BIO-NMD project.^43^

The recognition of EBB contribution has been usually recorded in the Acknowledgements or in the Materials and Methods sections of scientific publications. The users agree to acknowledge the role of the EBB facilities in the relevant publications by signing MTAs that are based on a model MTA agreed among EBB biobanks and their host institutions. The papers acknowledging the EBB contribution were determined either by direct communication of the EBB users, or by customized searches, for example, through Google Scholar.

EBB partners firmly recognize the importance of assessing the impact of bioresources and of facilitating their traceability. Indeed, some EBB partners (M Filocamo, J-H Di Donato) are currently contributing to the development of BRIF (Bioresource Research Impact Factor), the tool to calculate the research impact of bioresources based on an algorithm and a unique digital resource identifier^44^ and, once the pilot studies will be completed, the EBB network is expected to contribute to BRIF implementation. In particular, to obtain BRIF, the EBB partners will submit to BRIF-Open Journal of Bioresources a marker paper which, by describing the main characteristics of their Biobank, will allow tracking bioresource use in the scientific literature.^45^

### Samples for research by for-profit organizations

To develop better drugs for patients with RDs (translational biomedical research), academic organizations and infrastructures need to cooperate with the pharmaceutical industry. In this regard, the network has been involved in a number of pharmaceutical company projects involving either searches for disease biomarkers or testing new therapeutic approaches. In particular, myoblasts and fibroblasts from Duchenne muscular dystrophy patients have been given to Prosensa to enable them to test exon-skipping approaches to therapy; cells fromNMD patients have been given to Santhera Pharmaceuticals to help them develop pharmaceutical products; and serum samples from muscular dystrophy patients have been provided to Summit and Pfizer for biomarker discovery and validation.

## Quality-management system

The setting up of common SOP's for sample collection, processing and storage, the standardization of samples access policies, the adoption of a common minimum data set and controlled terminologies necessary to build the EBB catalogue were the first approach towards a QC system. A further step was taken by the EBB network in 2009 through the development of a satisfaction questionnaire to be sent to the users, for checking the quality of the services provided by the EBB biobanks. During years 2009–2010, each partner sent the questionnaire to the users who, once had the document filled in, sent their feedback anonymously to EURORDIS. The evaluation of the questionnaires received revealed that the users were overall either ‘very satisfied' or ‘satisfied' with the service, and that EBB was seen as an essential service for scientists involved in research on RD who would otherwise have no access to these samples. Because of the low response rate (19/54) the value of the survey remained limited; however, the users were satisfied with the provided service and in only 11% of cases encountered problems with the received samples; 84% of the responders confirmed that samples were essential for their experiments.

A reflexion process regarding quality system certification of the network and/or individual biobanks of the network began at the EBB meeting in 2010. During the 9th annual meeting, the network moved forward with a tutorial to discuss the requirements to be fulfilled if a biobank wishes to assess its current quality management system (QMS), with a view to upgrade it and move towards ISO certification. A QC questionnaire, listing the QMS requirements, was developed and used as the basis of the tutorial. On he basis of the QC questionnaire, the network planned further actions, feasible without additional funding, in order to acquire the ISO standards. These included implementation of self-assessment tools, harmonization of samples, and data processing through common SOPs, and adoption of best practice guidelines and recommendations published by OECD, NCI, and ISBER. Certification and/or formal accreditation in conformity with the EU ISO standards remain the ultimate goal.

## Future developments and challenges

The legal and regulatory frameworks that apply to this area are still fragmented, with variation of practice across the different areas of medical research.^46,^^47^ Biobanks have to live with these frameworks and practices, which have raised a number of complex issues for society. For example, next-generation sequencing techniques pose important issues in relation to the management of incidental findings.^48, 49^ Although tools aimed at facilitating data and sample sharing are being developed,^48^ privacy regulations and requirements vary between countries, rendering sample transfer between countries problematic. In addition, issues of communication between RD patient registries and RD biobanks still need to be addressed. A related problem is that different names are often used for a single disease creating difficulties when searching by disease. Other issues facing biobanking are the lack of standards, agreed vocabulary, common data elements, and best practices for collecting data and processing samples. An accreditation and evaluation system to recognise biobanks that provide high-quality samples, and reward and acknowledge scientists who establish and maintain biobanks, should be established. In this regard, BRIF, the recently proposed quantitative parameter to assess the use of bioresources by the biomedical community, will represent a starting point for biobank recognition.^44^ Another important issue in RD biobanking is sustainability. Clearly, the pharmaceutical industry has little interest in funding small RD biobanks that contain and exchange limited numbers of samples. Specific funding for RD biobanks is therefore essential. Finally, very few biobanks are concerned solely with RD samples, whereas some collections make material available only for specific types of studies and not others. In particular, biomaterials collected in natural history studies and clinical trials should be deposited in biobanks (with a broad consent allowing secondary use of the samples). Currently, most samples collected in these studies are ‘private' to these studies/investigators and cannot be shared (and often get destroyed at the end of the study). In this regard, the promotion of collaborations between biobanks and patient associations, in addition to helping gather more RD samples and associated data and make them available to researchers, could better address ethical and legal challenges because of the solidarity-based nature of the underlying agreements.^50^

Recently, an application sent by the EBB scientific coordinator to RD-Connect's executive committee, expressing EBB's interest in becoming a partner of the RD-Connect platform, received formal approval. RD-Connect's objective in biobanking is to provide access to RD biobanks that collect and provide standardised, quality-controlled biomaterials. In this regard, a dynamic, updated, searchable catalogue of biological samples linked to clinical data from patient registries and to patients' ‘omics' data will represent RD-Connect's major output.^51^ In this context, EBB's main aim is to contribute expertise to promote high professional standards and best practices in RD biobanking and implement the integration with RD patient registries. The RD-Connect project was developed under IRDiRC (International Rare Disease Research Consortium), a European Commission and US National Institute of Health initiative to coordinate research funding on RDs, and RD-Connect activities and policies are interconnected with those of IRDiRC. Therefore, EBB is providing work and expertise under the IRDiRC umbrella. Moreover, EBB partners are involved in IRDiRC biobanking-related programmes and activities, in particular through the IRDiRC working groups on biobanking (V Karkagi, H Lochmüller, M Mora), and on registries and natural history of diseases (M Posada) and the interdisciplinary science committee (H Lochmüller). The EBB contribution to the challenges faced by biobanking is therefore extending beyond Europe and, hopefully, provides contributions to biobanking on a global level.

## Conclusions

Over the past decade, the scale of biobanking activities, in terms of the quantity of samples and data archived, the range of diseases covered, and the institutions involved, have expanded markedly. Biobanks are embedded in complex networks of research collaborations that span regions and countries. However, most European citizens have never heard of biobanks nor do they know of their importance in research on RDs. Furthermore, the legal and regulatory frameworks that apply to this area are fragmented. The EBB has long been the only network seeking to confront ethical, legal, and social issues related to RD biobanking across European countries. In the absence of a unified regulatory framework, but by seeking professional guidance and applying professional values and culture, the EBB has been able to progress and contribute to the development of regulations as well as to establishing a basis for international cooperation in RD biobanking. Finally, EBB and biobanks in general need to overcome numerous challenges in order to achieve their full potential as essential aids to RD research. These include the following: lack of harmonization; lack of biomaterial and data sharing; lack of recognition; and lack of sustainability. The EBB, with its long experience in international biobanking, will continue to be a key global resource for life sciences research, drug development, and healthcare.

## Figures and Tables

**Table 1:** EBB members

*Member*	*Short name*	*Contact*	*Role*	*Status*
European Organization for Rare Diseases	EURORDIS	Fabrizia BIGNAMI^a^, Anna KOLE	Administrative coordinator 2003–2011	Founder
Association Française contre les Myopathies	AFM	Cécile JAEGER^b^^,^^c^, Thomas VOIT	EBB scientific coordinator 2003–2005, BB director	Founder
Istituto Nazionale Neurologico Carlo Besta	NEUMD-INNCB	Marina MORA	Current EBB scientific coordinator, BB Director	Founder
Fundación para la Cooperación y Salud International Carlos III—ISCIIII	Fundación CSAI Carlos III—ISCIII	Manuel POSADA DE LA PAZ	BB Director	Founder
Généthon III	Généthon	Jeanne-Hélène DI DONATO^c^^,^^d^, Saafa SAKER	BB Director	Founder
Centre de Génétique Humaine UCL (Université Catholique Louvain)	LOUVAIN	Christine VERELLEN-DUMOULIN	BB Director	Founder, retired after 1 year
University of Ljubljana, Medical Faculty	University of Ljubljana	Marija MEZNARIC	BB Director	Founder
The University of Malta	UOM	Dorita GALEA^c^, Alex FELICE	BB Director	Founder
Muscle Tissue Culture Collection (MTCC) at the Friedrich-Baur-Institut of the Ludwig-Maximilians-Universität, Munich	MTCC	Hanns LOCHMÜLLER^c^, Peter SCHNEIDERAT	BB Director	Founder
Second University of Naples	SUN	Luisa POLITANO	BB Director	Founder
Fodor József national Center for Public Health, Budapest	NCPH	Veronika KARCAGI	BB Director	Founder
Ospedale Maggiore Policlinico IRCCS, University of Milan, Dpt. of Neurological Sciences	NMUNIT—UNIMIOM	Maurizio MOGGIO	BB Director	Founder
University of Padova, Department of Neurological and Psychiatric Sciences	NMTB	Corrado ANGELINI^c^, Elena PEGORARO	BB Director	Founder
Bio Expertise Technologies	B.E.T	Jean-Claude LAURENT	SME for cell culture development	Founder, retired at the end of EC funding
Université Joseph Fourier—Grenoble 1	UJF	Olivier COHEN	Database and website	Founder, retired at the end of EC funding
TEAMLOG SA	TEAMLOG	Christophe GUITART ARNAU	Database and website	Founder, retired at the end of EC funding
Bank for the Diagnosis and Research of Movement Disorders, Istituto Neurologico Carlo Besta	MDB-INNCB	Barbara GARAVAGLIA	BB Director	Joined in 2008
Bank of the National Laboratory for the Genetics of Israeli Populations	NLGIP	David GURWITZ	BB Director	Joined in 2009
MRC Centre for Neuromuscular Diseases BioBank, London	CNMD-BBL	Francesco MUNTONI, Diana JOHNSON	BB Director	Joined in 2010
MRC Centre for Neuromuscular Diseases BioBank, Newcastle	CNMD-BBN	Hanns LOCHMÜLLER, Mojgan REZA	EBB scientific coordinator 2005–2011, BB Director	Joined in 2010
Quebec Myotonic Dystrophy Biocatalog	QMDB	Jack PUYMIRAT	BB Director	Joined in 2010
Cell line and DNA Biobank from patients affected by Genetic Diseases, Istituto Giannina Gaslini, Genova	IGG-GB	Mirella FILOCAMO	TNGB Coordinator, BB Director	Joined in 2012
Galliera Genetic Bank, Ospedali Galliera, Genova	GGB	Chiara BALDO	BB Director	Joined in 2012
Cell lines and DNA Bank of Rett syndrome, X-linked mental retardation and other genetic diseases, University of Siena	biobankUNISI	Alessandra RENIERI	BB Director	Joined in 2012
Parkinson Institute Biobank, Istituti Clinici di Perfezionamento, Milano	BPI	Stefano GOLDWURN	BB Director	Joined in 2012
Genomic Disorders Biobank IRCCS Casa Sollievo della Sofferenza, S Giovanni Rotondo	GDDB	Giuseppe MERLA	BB Director	Joined in 2012
Fondazione Telethon	FTELE	Lucia MONACO, Marco CRIMI	Administrative coordinator	Joined in 2012
Dipartimento Ligure di Genetica, Genova	DLG	Franca DAGNA-BRICARELLI	TNGB Coordinator Emeritus	Joined in 2012

a Now at GSK, London.

b Now at Hôpital Foch, Suresnes.

c Former Biobank Director.

d Now at 3 C-R (Conseil et expertise pour les biobanques).

**Table 2:** EBB Work package (WP) organization

*WP N.*	*WP title*	*Achievements*	*Responsible participants (N)*^a^
1	Coordination and organisation of the network	Elaborated the EBB Charter Developed policies for approval of projects for which the biological samples are requested.	**2-**1-3-4-5-9-13-15
2	Technical and quality issues for DNA	Worked to harmonize SOPs for DNA sample collection	**5-**3-4-6-8-10-11-12-13
3	Technical and quality issues for tissue	Worked to harmonize SOPs for tissue sample collection	**13-**2-7-10-12–14
4	Technical, quality issues, and development for cell culture	Worked to harmonize SOPs for cell culture sample collection	**3-**5-6-9-12-13-14
5	Ethical issues	Harmonized the donor consent form. Addressed confidentiality and data protection issues. Developed policies for the collection and use of the biological material for research and access to samples, including respect of non-patrimoniality	**2-**1-4-5-11-12-15
6	Database and website	Developed a website and a catalogue	**15-**4-5-16
7	Communication	Improved communication with donors/patients on the use of collections and the outcomes of the research projects thorough website, information leaflets and personal communication to the patients	**1**-6-7-9-10
8	Training and technology transfer	Technology and know how transfer to partners and users through training courses and through individual e-mail and phone counselling.	**9-**3-8-10-11

a Work package leader listed first.
